# Mitigation of Marine Dinoflagellates Using Hydrogen Peroxide (H_2_O_2_) Increases Toxicity towards Epithelial Gill Cells

**DOI:** 10.3390/microorganisms11010083

**Published:** 2022-12-28

**Authors:** Jorge I. Mardones, Ana Flores-Leñero, Marco Pinto-Torres, Javier Paredes-Mella, Sebastián Fuentes-Alburquenque

**Affiliations:** 1 Centro de Estudios de Algas Nocivas (CREAN), Instituto de Fomento Pesquero (IFOP), Puerto Montt 5501679, Chile; 2 CAICAI Foundation, Puerto Varas 5550000, Chile; 3 Centro de Investigación en Dinámica de Ecosistemas Marinos de Altas Latitudes (IDEAL), Valdivia 5110566, Chile; 4 Centro de Investigación en Recursos Naturales y Sustentabilidad, Universidad Bernardo O’Higgins, Santiago 8370993, Chile; 5 Departamento de Matemáticas y Ciencias de la Ingeniería, Facultad de Ingeniería Ciencia y Tecnología, Universidad Bernardo O’Higgins, Santiago 8370993, Chile

**Keywords:** harmful algal blooms (HABs), phytoplankton, reactive oxygen species (ROS), lipid peroxidation, toxic aldehydes, RTgill-W1 cell line

## Abstract

Hydrogen peroxide (H_2_O_2_) has been shown to efficiently remove toxic microalgae from enclosed ballast waters and brackish lakes. In this study, in vitro experiments were conducted to assess the side effects of mitigating toxic and non-toxic dinoflagellates with H_2_O_2_. Five H_2_O_2_ concentrations (50 to 1000 ppm) were used to control the cell abundances of the toxic dinoflagellates *Alexandrium catenella* and *Karenia selliformis* and the non-toxic dinoflagellates *Lepidodinium chlorophorum* and *Prorocentrum micans.* Photosynthetic efficiency and staining dye measurements showed the high efficiency of H_2_O_2_ for mitigating all dinoflagellate species at only 50 ppm. In a bioassay carried out to test cytotoxicity using the cell line RTgill-W1, control experiments (only H_2_O_2_) showed cytotoxicity in a concentration- and time- (0 to 24 h) dependent manner. The toxic dinoflagellates, especially *K. selliformis*, showed basal cytotoxicity that increased with the application of hydrogen peroxide. Unexpectedly, the application of a low H_2_O_2_ concentration increased toxicity, even when mitigating non-toxic dinoflagellates. This study suggests that the fatty acid composition of toxic and non-toxic dinoflagellate species can yield toxic aldehyde cocktails after lipoperoxidation with H_2_O_2_ that can persist in water for days with different half-lives. Further studies are needed to understand the role of lipoperoxidation products as acute mediators of disease and death in aquatic environments.

## 1. Introduction

Some coastal regions of the world are experiencing an increase in harmful algal blooms (HABs), especially under the ongoing climate change scenario [1]. These HABs have exerted significant negative impacts on human and environmental health, tourism, economies and worldwide aquaculture production [2,3]. These deleterious impacts produced by HABs present an important challenge to the institutions responsible for the management of coastal resources, resulting in the implementations of a wide variety of mitigation strategies, among which the use of chemicals, such as hydrogen peroxide (H_2_O_2_), stands out.

Hydrogen peroxide, along with ion superoxide (O_2_^−^) and hydroxyl radicals (OH·), is a reactive oxygen species (ROS) that is intermediate in the four-electron reduction of oxygen to water [4]. Since the discovery of the ubiquitous antioxidant enzyme superoxide dismutase (SOD) [5], it has been well-recognized that all oxygen-metabolizing organisms produce ROS. Among aquatic organisms, HAB species present the highest rates of extracellular ROS production [6]. For instance, the raphidophyte *Chattonella marina* [7] and the dinoflagellates *Margalefidinium polykrikoides* [8] and *Alexandrium catenella* [9] are well-known ROS producers. Because H_2_O_2_ (as a component of ROS species) occurs naturally in the environment and decays to oxygen and water as a result of both biological and abiotically driven chemical reactions [4,10], it has been widely used to mitigate HABs, mainly in freshwater bodies but also in brackish environments [11,12]. 

As a strong oxidant, H_2_O_2_ is known for its disinfectant characteristics, as well as its effect on microalgae by inhibiting photosynthetic activity due to damage to photosystem II [13,14]. It is applied directly to the water body, and its efficiency in removing microalgae is mainly examined via direct cell counts and/or by monitoring fluorescence as a proxy for the presence of microalgae pigments [13]. Among HAB species, cyanobacteria, such as *Microcystis aeruginosa*, are known to be ten times more sensitive to hydrogen peroxide than diatoms and green algae [11,15]. In general, low concentrations of H_2_O_2_ have been shown to be effective against a variety of cyanobacteria species [13,16,17]. For instance, H_2_O_2_ was applied for the first time to an entire lake in 2009; 2 mg L^−1^ was applied to selectively inhibit the growth of *Planktothix agardhi* in Lake Koetshuis, the Netherlands [11], and 3 mg L^−1^ was applied to remove the same species from Lake Anita Louise, Maryland, the USA [18]. In other cases, higher amounts of H_2_O_2_ (44–95 mg L^−1^) have been used to remove all microcystins and two-thirds of cyanobacteria from a wastewater pond [19]. However, effective H_2_O_2_ concentrations required to effectively eradicate toxic dinoflagellates are apparently case-specific. Burson et al. [12] showed that the addition of 50 mg H_2_O_2_ L^−1^ was sufficient to kill the pellicle cysts and vegetative cells of *Alexandrium ostenfeldii* in the brackish Ouwerkerkse Kreek, the Netherlands. In Kagoshima Bay, Japan, 30 mg L^−1^ was needed for rapid declines of *Cochlodinium* sp., and in parallel laboratory cyst experiments, >10 mg L^−1^ and 10–100 mg L^−1^ were required to severely depress the excystment of *Alexandrium catenella* and *Polykrikos schwartzii*, respectively [20]. In other cases, concentrations of up to 5000 mg H_2_O_2_ L^−1^ have been required to eliminate the resting cysts of the paralytic shellfish toxin (PST) producer *Gymnodinium catenatum* from ships´ ballast waters [21].

Besides its effectiveness for HAB mitigation, several concerns arise when using hydrogen peroxide for its ecosystem effects. For instance, (1) a variety of studies have shown the synergistic effect of ROS species and microalgae polyunsaturated fatty acids (PUFAs) in producing cytotoxic byproducts [22,23,24,25]; (2) climate change is rapidly increasing the growth niche of toxic flagellates by enhancing water stratification [26], resulting in the requirement of effective and environmentally safe HAB mitigation strategies; and (3) sewage treatment and the control of sea lice in the salmon industry use high amounts of H_2_O_2_, which can interact with lipids from different biological matrices, including phytoplankton. Because of these concerns, the main goal of this study was to determine if the reported low doses of H_2_O_2_ (50 ppm) used to mitigate toxic dinoflagellates in open waters could potentially produce environmental side effects that affect the local marine biota. We used a cell-based bioassay as a proxy and included non-toxic dinoflagellate species to assess the putative synergistic reaction of H_2_O_2_ with other organic molecules (not toxins) that increase toxicity with potential ecosystem effects.

## 2. Materials and Methods

### 2.1. Culture Conditions

#### 2.1.1. Marine Dinoflagellates

Microalgae strains were isolated from different regions of the Patagonian fjords in southern Chile. The toxic dinoflagellates *Alexandrium catenella* (CREAN_AC16 strain) and *Karenia selliformis* (CREAN_KS02) were isolated from the Aysén Region in 2019 and 2018, respectively. The non-toxic dinoflagellates *Lepidodinium chlorophorum* (CREAN_LC01 strain) and *Prorocentrum micans* (CREAN_PM02) were isolated in 2020 from the Los Lagos Region from the localities of Yates and the Moraleda Channel [27], respectively. All cultures were maintained in the CREAN-IFOP algal collection in Puerto Montt, Chile, in L1 sterile medium at 15 °C and at a salinity of 33 under 100 μmol photon m^−2^ s^−1^ and a 18:6 h light:dark cycle.

#### 2.1.2. Epithelial RTgill-W1 Cell Line

A detailed description of the method used for the gill cell line culturing is provided by Mardones et al. [28]. The RTgill-W1 cell line (CRL-2523) was acquired from the American Type Culture Collection (ATCC, Manassas, VA, USA). The cell line was maintained in the dark at 19 °C in 25 cm^2^ culture-treated flasks containing Leibovitz’s L-15 medium (Sigma, St. Louis, MO, USA, L1518) supplemented with an antibiotic–antimycotic mixture (Sigma, A5955, St. Louis, MO, USA), comprising penicillin (10,000 units mL^−1^), amphotericin B (25 mg mL^−1^) and streptomycin (10 mg mL^−1^), and 10% (*v*/*v*) fetal bovine serum (FBS) (Sigma, 12003C, St. Louis, MO, USA). For sub-culturing twice per week, TrypLE™ Express (Gibco™, Billings, MT, USA) solution was used to detach the cells from the bottom of the flasks, and L-15 medium was added at a ratio of 1:2.

### 2.2. Culture Chronic and Acute Effect of H_2_O_2_ on Toxic Dinoflagellates

To determine the effects of five different concentrations (50, 100, 200, 500 and 1000 ppm) of H_2_O_2_ (30% *v*/*v*, Sigma-Aldrich, St. Louis, MO, USA) on the toxic dinoflagellates, the cells of *A. catenella* and *K. selliformis* (1000 mL^−1^) were inoculated in triplicate into sterile 50 mL flasks containing 40 mL of seawater with L1 culture medium. Each H_2_O_2_ treatment was assessed using a crossed factorial design with two different salinities (25 and 33) and two different temperatures (12 and 18 °C). The experiment was carried out for 1 h, with samples taken immediately after exposure. The chronic effects of H_2_O_2_ on the dinoflagellate cultures were determined instantly after sampling based on the photosynthetic efficiency (PE) of the cells, and acute effects were determined based on direct observations of dinoflagellate cell viability (DCV) under an inverted microscope.

#### 2.2.1. Photosynthetic Efficiency (PE) Measurement

The physiological responses of the toxic dinoflagellates to the different H_2_O_2_ concentrations were assessed by measuring the maximal photosynthetic efficiency (PE) (F_v_/F_m_) using a fast repetition rate fluorometer (FRRf, Chelsea Technologies Group, London, UK). The PE is described by Van Kooten and Snel [29] as follows:

(F_v_/F_m_) = (F_m_ − F_o_)/(F_m_) (1)

where F_o_ is the minimal fluorescence in light, F_m_ is the maximal fluorescence in light, and F_v_ is the variable fluorescence in light. The measuring protocol of the FRRf was set to an acquisition sequence of 20 saturation flashes in 1 min, 20 relaxation flashes and 10 m/s sleep time between acquisitions. The flash duration was 0.65 µs. This sampling protocol was found to better characterize the fluorescence response (i.e., saturation curve fitting) of the dinoflagellate cultures in preliminary tests. F_v_/F_m_ was measured 1 h after using hydrogen peroxide in the control and treatment samples. The calculation of the dinoflagellate PE % of the control is described as follows:

Dinoflagellate PE (% of control) = (PET/PEC) × 100(2)

where PET corresponds to the photosynthetic efficiency of the experimental treatments, and PEC corresponds to the photosynthetic efficiency of the control treatments.

#### 2.2.2. Dinoflagellate Cell Viability (DCV) Test

The acute effect of H_2_O_2_ on dinoflagellate cell viability was assessed using Neutral Red (NR) following Onji et al. [30]. NR (Sigma-Aldrich, St. Louis, MO, USA) is a cationic dye that is able to permeate the plasmatic membrane and accumulate in the lysosomes of viable cells. Viable cells turn red. To test cell viability, a filtered (0.22 µm) stock solution was prepared by dissolving NR in ethanol 95% at a final concentration of 1% *m*/*v*. Later, the stock solution was dissolved in seawater (1:5 *v*/*v*) at a final concentration of 0.2% *m*/*v*. The final solution was added to 2 mL of each sample at a ratio of 1:10 *v*/*v* and incubated for 15 min at room temperature and under light. A Sedgwick Rafter chamber was used for microalgae cell counting to estimate cell viability. The DCV was calculated as follows:

DCV (% of control) = (LTC/LCC) × 100(3)

where LCT corresponds to the number of live cells after the experimental treatments, and LCC corresponds to the number of live cells after the control treatments.

### 2.3. RTgill-W1 Bioassay to Test Environmental Cytotoxic Effects

#### 2.3.1. Marine Temporal Effect of H_2_O_2_ on the RTgill-W1 Cell Line

To assess the persistence of hydrogen peroxide toxicity in seawater, the RTgill-W1 cell line was exposed to six H_2_O_2_ concentrations (0, 50, 100, 200, 500 and 1000 ppm) for 1 h in the dark. The cytotoxic effects of all treatments were measured at 0, 4 and 24 h after the preparation of the dilutions. After exposure, gill cell viability was measured as described in Section 2.4.

#### 2.3.2. Combined Effects of H_2_O_2_ and Toxic and Non-Toxic Dinoflagellates on Gill Cells

To test the combined effects of six concentrations of H_2_O_2_ (0, 50, 100, 200, 500 and 1000 ppm) and the toxic dinoflagellates *A. catenella* and *K. selliformis* and the non-toxic dinoflagellates *P. micans* and *L. chlorophorum*, the cells of the factorial combination of each culture (1000 mL^−1^) were separately inoculated into each H_2_O_2_ treatment concentration. The toxic dinoflagellates were cultured at salinities of 25 and 33 and at 12 and 18 °C, and the non-toxic dinoflagellates were cultured at 15 °C at a salinity of 33. For comparison purposes in the experiment using the non-toxic dinoflagellates, the toxic dinoflagellate *A. catenella* (cultured under the same salinity and light conditions) was added to the experimental setup as a positive control treatment. All treatments were later filtrated (0.22 µm) and inoculated in quadruplicate in 96-well plates containing RTgill-W1 cells attached to the bottom of the wells, and they were kept for 1 h in the dark at 19 °C. After exposure, gill cell viability was measured as described in Section 2.4. 

### 2.4. Gill Cell Viability Endpoint

The RTgill-W1 bioassay has been widely used to detect cytotoxic activity in marine waters [6]. The assay estimates cell viability based on fluorescent indicator dyes when exposed to toxicants expressed as a percentage of unexposed control. Briefly, gill cell viability was determined using the indicator dye AlamarBlue 5% (DAL1025, Invitrogen, Waltham, MA, USA) diluted in phosphate-buffered saline (PBS). One hundred μL of the AlamarBlue solution was added to all cell-seeded wells and incubated for 1 h in the dark. AlamarBlue (non-fluorescence resazurin) enters the cells and is converted to the fluorescence resorufin by cytoplasmatic, mitochondrial or microsomal oxidoreductases. A decline in AlamarBlue fluorescence indicates a reduction in cellular metabolism (low GCV). A microplate reader (FLUOstar Omega, BMG Labtech 415-2871, Ortenberg, Germany) was used to measure the fluorescence of the dye with excitation and emission filters of 540 and 590 nm, respectively. Gill cell viability (GCV) is shown as the response percentage of the experimental treatments relative to that of the control treatments (% of control).

### 2.5. Statistical Analysis

The effects of the hydrogen peroxide concentration, species, salinity, temperature and time of exposure on the response variables, namely, (1) gill cell viability (GCV), (2) dinoflagellate cell viability (DCV) and (3) photosynthetic efficiency (PE), were assessed using an analysis of variance (ANOVA). All factors were evaluated as discrete variables, except for “concentration”, which was assessed as a continuous variable. The ANOVAs were performed in a generalized lineal model (GLM) framework fitting a negative binomial residual distribution model. For significant effects, a Tukey’s HSD multiple comparison test was used. In each case, the null statistical hypotheses were rejected under a significance level (α) of 0.05. All analyses were performed using R software version 4.1.2 (R Core Team, 2017), and the GLM was fitted using the R package “lme4” version 1.1–31 [31].

## 3. Results

### 3.1. Effect of H_2_O_2_ Concentration, Species, Temperature and Salinity on PE, DCV and GCV

**Figure 1:**
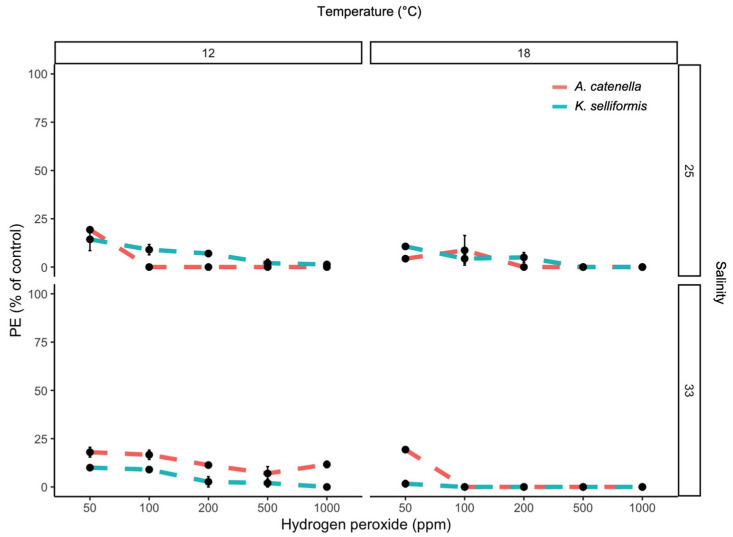
Photosynthetic efficiency (PE) (F_v_/F_m_) of the toxic dinoflagellates *A. catenella* and *K. selliformis* exposed to five hydrogen peroxide concentrations under two salinity and two temperature conditions.

**Figure 2:**
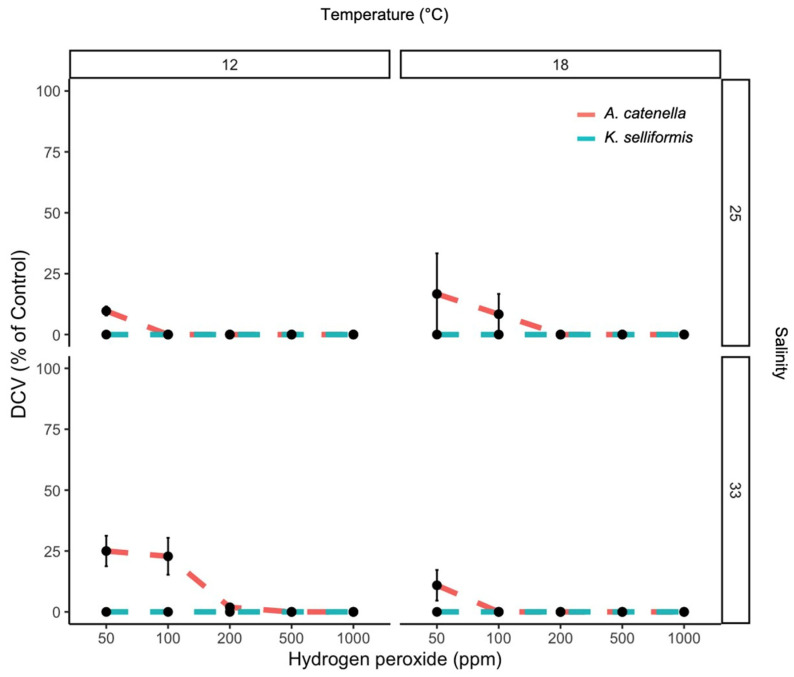
Dinoflagellate cell viability (DCV) of the toxic dinoflagellates *A. catenella* and *K. selliformis* exposed to five hydrogen peroxide concentrations under two salinity and two temperature conditions.

**Figure 3:**
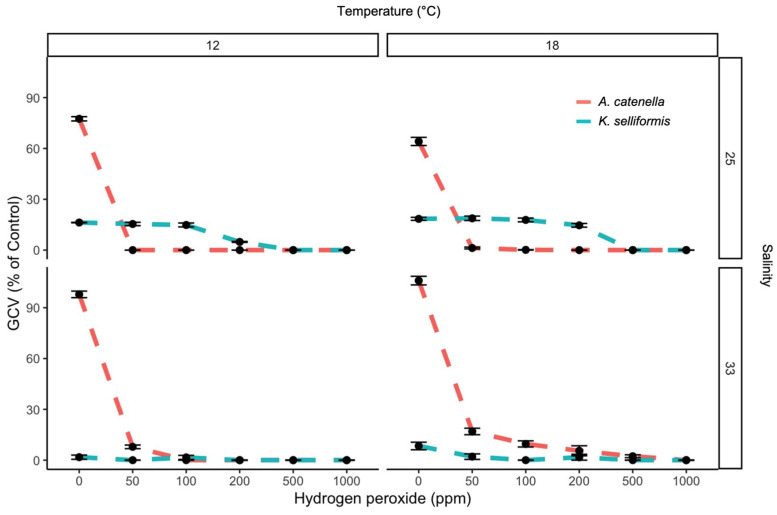
RTgill-W1 cell viability (GCV) after 1 h exposure to five hydrogen peroxide concentrations combined with 1000 cells mL^−1^ of the toxic dinoflagellates *A. catenella* and *K. selliformis* under two salinity and two temperature conditions. Symbols represent the mean, and error bars represent the standard deviation of cell viability from quadruplicate measurements.

The PE and DCV showed low values, even when the H_2_O_2_ concentrations were minimal. The highest PE (12 %) was observed at 50 ppm of H_2_O_2_, and the lowest PE (1.6%) was observed at 1000 ppm (Figure 1). The maximum and minimum DCV measurements were also achieved at 50 and 100 ppm of H_2_O_2_, with values of 6.4 and 0%, respectively (Figure 1 and Figure 2). The PE was significantly affected by the peroxide concentrations (negative correlation) and temperature (*p* < 0.05). Salinity and species did not show effects (*p* > 0.05). The DCV was significantly affected by the peroxide concentrations, temperature, species and salinity (*p* < 0.05). The GCV was significantly affected by the peroxide concentrations (negative correlation), temperature and species (*p* < 0.05) compared to the control (100% ± 3% cell viability). The toxic dinoflagellates *K. selliformis* and *A. catenella* severely affected the gill cells in the control treatment (without H_2_O_2_), with GCV values lower than 50% (LC_50_) in *K. selliformis* (Figure 3). The addition of H_2_O_2_ to the toxic dinoflagellate cultures increased the cytotoxicity to the gill cells, with GCV values of 0 and 7.7 at 1000 and 50 ppm, respectively. 

### 3.2. Effects of H_2_O_2_ Concentration and Time of Exposure on GCV

**Figure 4:**
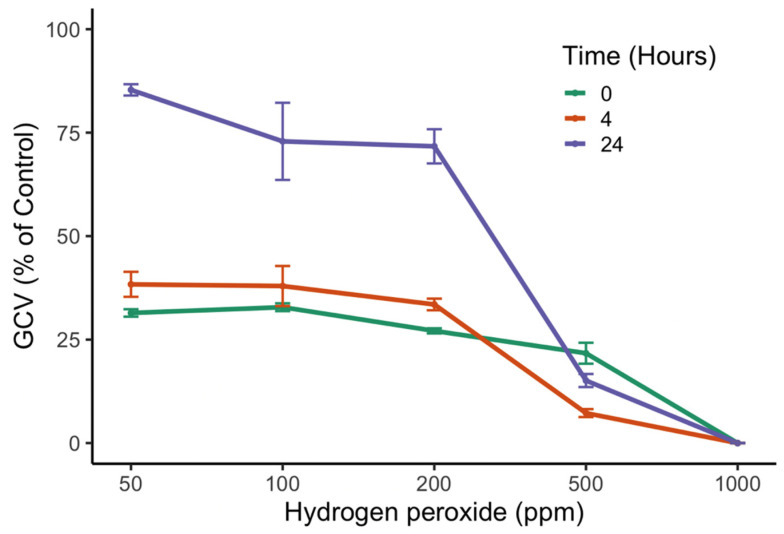
RTgill-W1 cell viability (GCV) after 1 h exposure to five hydrogen peroxide concentrations. Measurements were carried out at 0, 4 and 24 h after H_2_O_2_ dilutions. Symbols represent the mean, and error bars represent the standard deviation of cell viability from quadruplicate measurements.

The peroxide concentrations and time of exposure significantly affected the GCV (*p* < 0.05). A posteriori Tukey test showed a negative response of the gill cells after 0 and 4 h of H_2_O_2_ exposure, with GCV values lower than 50% (LC_50_). However, after 24 h of treatment, viability was recovered at the lower peroxide concentrations, 50 and 200 ppm, with values of 80% and 75%, respectively. Gill cells exposed to treatments of 500 and 1000 ppm showed CGV values of 24% and 0%, respectively (Figure 4).

### 3.3. Synergistic Effect of H_2_O_2_ and Toxic and Non-Toxic Dinoflagellates on GCV

The toxic dinoflagellates *K. selliformis* and *A. catenella* severely affected the gill cells in the control treatment (without H_2_O_2_), with GCV values lower than 50% (LC_50_) for *K. selliformis* (Figure 4). The GCV was significantly affected by the peroxide concentrations (negative correlation), temperature and species (*p* < 0.05). The addition of H_2_O_2_ to the toxic dinoflagellate cultures increased the cytotoxicity to the gill cells, with GCV values of 0 and 7.7 at 1000 and 50 ppm, respectively.

**Figure 5:**
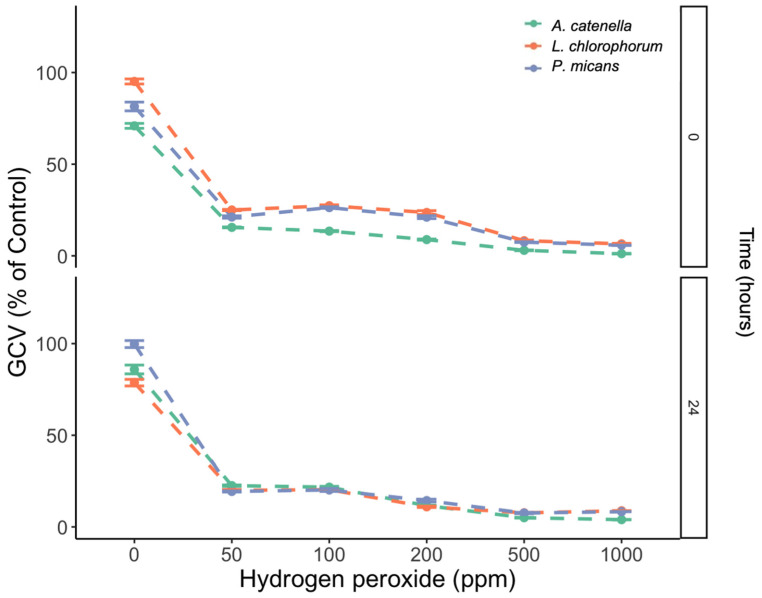
RTgill-W1 cell viability (GCV) after 1 h exposure to five hydrogen peroxide concentrations combined with 1000 cells mL^−1^ of the toxic dinoflagellate *A. catenella* and the non-toxic dinoflagellates *L. chlorophorum* and *P. micans*. Measurements were carried out at 0 and 24h after H_2_O_2_ + dinoflagellate mixing. Symbols represent the mean, and error bars represent the standard deviation of cell viability from quadruplicate measurements.

An increase in H_2_O_2_ concentration resulted in significant attenuation of the GCV (*p* < 0.05), with GCV values of 75 % (control without peroxide) and 0% (1000 ppm peroxide). Despite the significant differences among species (*p* < 0.05), the addition of H_2_O_2_ reduced the GCV below the LC_50_ in both the toxic and non-toxic dinoflagellate treatments. After 24 h of exposure to H_2_O_2_ and the dinoflagellates, the GCV remained as low as that at 0 h (*p* > 0.05) (Figure 5).

## 4. Discussion

The present study investigated the potential deleterious effect of H_2_O_2_ on epithelial gill cells as a proxy for environmental safety risk after its application in marine waters to cease toxic and non-toxic dinoflagellate blooms.

### 4.1. Mitigation of Dinoflagellates Using H_2_O_2_

This study shows that the mitigation of toxic armored (*A. catenella*) and unarmored (*K. selliformis*) dinoflagellates using hydrogen peroxide is an effective method, even when using doses as low as 50 ppm (Figure 1 and Figure 2). These in vitro results are in line with those found by Burson et al. [12], where 50 ppm of H_2_O_2_ was efficient to mitigate an *A. ostenfeldii* bloom and its paralytic toxins in the brackish Ouwerkerkse Kreek, the Netherlands. Even a lower dose of 30 ppm has been found to be effective in mitigating the ichthyotoxic dinoflagellate *Cochlodinium* sp. in Japan [20]. A key question is whether abiotic variables, such as temperature and salinity, can affect the effectiveness of H_2_O_2_ in mitigating toxic dinoflagellates or other HAB groups. The experiments in this study showed that temperature influenced the effectiveness of H_2_O_2_ on the PE response of the dinoflagellates (chronic effect), and both temperature and salinity affected dinoflagellate cell viability when exposed to hydrogen peroxide (acute effect). It has been shown that salinity and temperature interact with H_2_O_2_ degradation in seawater [32], but these two variables can also alter microalgae cell permeability, affecting osmotic processes [33]. Thus, careful consideration must be taken when using H_2_O_2_ for HAB mitigation in highly variable aquatic scenarios. 

The Chilean dinoflagellates *A. catenella* and *K. selliformis* have been shown to be cytotoxic to the RTgill-W1 cell line [9,34]. One possible cytotoxic mechanism in both dinoflagellates is the synergistic reaction between long-chain PUFAs (>20 carbons) and ROS. However, both species produce other phycotoxins that can exert cytotoxic effects. Although true phycotoxins were not measured in this study, previous studies carried out by our research group have shown that the paralytic toxin analogs produced by *A. catenella* have low cytotoxic effects, whereas the brevenal-like compounds produced by *K. selliformis* have proven to be extremely cytotoxic. Despite the higher toxicity of *K. selliformis* towards the gill cells, a strong synergistic effect between hydrogen peroxide and the dinoflagellates was observed. This effect was surprisingly strong for *Alexandrium*, where the gill cell viability dropped from 93 to 60% with *Alexandrium* only and from 93 to 0% with *Alexandrium* + 50 ppm H_2_O_2_. This effect was also observed in previous studies, where lysed cells generated ROS, which increased toxicity towards gill cells [9]. Hydrogen peroxide, besides lysing dinoflagellate cells, (1) may oxidize some of the cellular components, probably yielding metabolites that boost *Alexandrium* toxicity, and/or (2) may interact with other toxic metabolites, such as paralytic shellfish toxins (PSTs). The oxidizing effect of H_2_O_2_ on PSTs has not been addressed to date, but the termination of the *A. ostenfeldii* bloom reported by Burson et al. [12] indicated that the concentration of PSTs was reduced after treatment with H_2_O_2_.

This study shows that the toxic effect of H_2_O_2_ on the gill cell line degrades rapidly after 24 h at concentrations lower than 200 ppm (Figure 4). Higher concentrations of >1000 ppm can persist longer in seawater. Some studies have reported half-lives of hydrogen peroxide ranging from hours to more than 7 days, with them being highly dependent on organic matter concentration [32,35]. Thus, the obvious concern rises: can peroxide trigger toxicity in non-toxic dinoflagellates? The experiments that followed included the non-toxic dinoflagellate species *Lepidodinium chlorophorum* and *Prorocentrum micans*, where H_2_O_2_ triggered a toxic effect at levels comparable to those observed for *A. catenella* and *K. selliformis* when exposed to the oxidizer (Figure 5). These results support the hypothesis that hydrogen peroxide can boost toxic metabolite generation when mitigating toxic and non-toxic dinoflagellate blooms.

### 4.2. Toxicity in Dinoflagellate Bloom Mitigation Using H_2_O_2_

Reactive oxygen species, including H_2_O_2_, can affect virtually any organic molecule susceptible to oxidation. Thus, ROS have a systemic effect on cells, with membrane fatty acids being one of the main targets of oxidation. Polyunsaturated fatty acid (PUFA) lipoperoxidation is a well-studied mechanism of oxidative stress, and it has been extensively studied in the clinical context for the formation of metabolites that can act as toxic or signaling molecules [36,37]. Oxidation can occur at different points of the molecule depending on the location of reactive sites (i.e., double bonds and functional groups). Thus, PUFA lipoperoxidation yields a cocktail of molecules depending on the locations of the unsaturations. For instance, ω6 PUFAs, such as linoleic and arachidonic acids, yield mainly 4-hydroxy-2*E*-nonenal (4-HNE), whereas ω3 PUFAs, such as docosahexanoic (DHA) acid, yield mainly 4-hydroxy-2*E*-henenal (4-HHE) [38,39]. Similarly, the oxidative degradation of any PUFA with more than two methylene-interrupted double bonds can yield malondialdehyde (MDA) [40]. For instance, the fatty acid composition of the Chilean *A. catenella* is dominated by ω3 PUFAs, with DHA (22:6ω3), octadecapentanoic (18:5ω3), stearidonic (18:4ω3), alpha-linoleic (18:3ω3) and eicosapentaenoic (20:5ω3) acids accounting for more than 50% of the fatty acids [9]. Since all the dominant PUFAs in this dinoflagellate have methylene-interrupted double bonds, it is probable that 4-HHE and MDA are the main aldehydes generated as lipoperoxidation products. 

**Figure 6:**
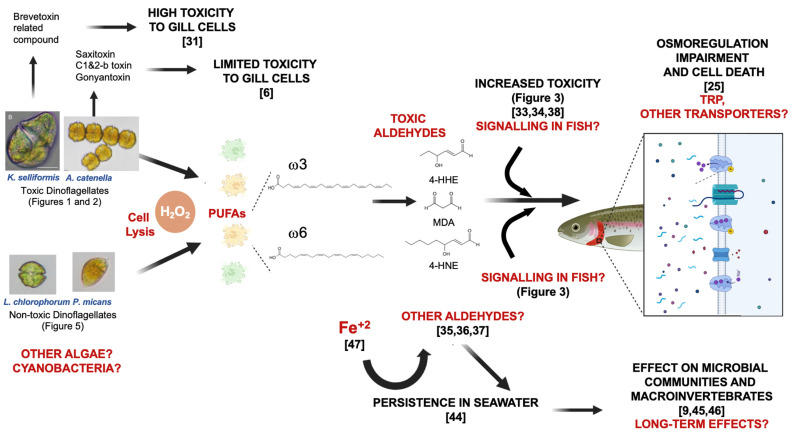
Proposed mechanism for cytotoxic byproduct generation after the use of hydrogen peroxide on dinoflagellate blooms. Aldehydes produced as a consequence of lipoperoxidation increase toxicity by impairing gill cell function.

Aldehydes react with the primary amines present in proteins or DNA. These covalent modifications impair the structure and function of biomolecules, which results in cytotoxicity and a loss of viability [37]. As membrane proteins are directly exposed to the cell environment, sensors and transporters (e.g., ions and glutamate) are two of the primary targets [36,41]. For instance, the transient receptor potential (TRP) cation channel superfamily that regulates several physiological processes is impaired by MDA, 4-HNE and 4-HHE [41]. Lysed *A. catenella* cells can impair osmoregulation via a net K^+^ efflux resulting in gill cell death [9,28]. Even though osmoregulation in other species, such as the model *Danio rerio* (zebrafish), is well-described, there is still knowledge gaps regarding the transporters involved [42]. In *Salmo salar* (Atlantic salmon), TRP transporters are expressed in several tissues, including the blood, spleen, kidney and gills [43]; thus, the involvement of this mechanism in osmoregulation in the rainbow trout RTgill-W1 cell line cannot be discarded. In Figure 6, this study proposes a ROS-mediated toxicity mechanism for gill cells in response to H_2_O_2_ usage in order to mitigate HABs.

Aldehydes are generated naturally by the breakdown of PUFAs in algae. For instance, arachidonic acid is largely accumulated in the microalgae *Lobosphaera incisa* [44], the brown algal kelp *Laminaria digitata* [45] and the dinoflagellate *Prorocentrum cordatum* [46]. Thus, the presence of these species represents an important potential source of aldehydes. Nevertheless, they are part of the volatile compounds produced under different environmental scenarios and related to defense mechanisms, such as oxylipin pathways [47]. The mitigation of algal or cyanobacterial blooms with H_2_O_2_, where populations can be as high as 10^9^–10^10^ cells m^−3^, may be an important source of toxic aldehydes that affect other species in the ecosystem. For example, the termination of blooms carried out in the Netherlands, the USA and Brazil had an effect on the whole microbial, phytoplankton, zooplankton and/or macroinvertebrate community composition [12,48,49]. One of these studies suggests that H_2_O_2_ treatment may not be an ideal mitigation approach in high-biomass ecosystems [48]. Surprisingly, none of these studies measured the generated metabolites, such as toxic aldehydes, before and/or after treatment. The fact that toxic aldehydes can persist in the water for days [47] and that the production can be increased by environmental parameters, such as the presence of iron ions [50] or other transition metals in the presence of H_2_O_2_, drives the need for further studies on the underlying mechanisms that boost their production in aquatic environments.

## 5. Conclusions

In this study, we show that the use of hydrogen peroxide for mitigating dinoflagellates enhanced the cytotoxicity in toxic dinoflagellate species but also caused toxicity when used in non-toxic dinoflagellates. We suggest that the fatty acid composition of microalgal blooming species can yield aldehyde cocktails under the oxidative stress produced by H_2_O_2_. Overall, further studies are needed to better understand the mechanisms underlying ROS-mediated toxicity in aquatic environments.

## Data Availability

Not applicable.
